# Inappropriateness of RNAlater to preserve *Caenorhabditis elegans* for RNA extraction

**DOI:** 10.1016/j.mex.2019.10.015

**Published:** 2019-10-18

**Authors:** Leming Jiang, Linyan Li, Ping Kang, Hai Yu, Shao-Ping Nie, Ming-Yong Xie, Joshua Gong

**Affiliations:** a State Key Laboratory of Food Science and Technology, Nanchang University, Nanchang, Jiangxi, 330047, China; b Guelph Research and Development Centre, Agriculture and Agri-Food Canada, Guelph, Ontario, N1G 5C9, Canada; c Hubei Key Laboratory of Animal Nutrition and Feed Science, Wuhan Polytechnic University, Wuhan, 430023, China

**Keywords:** Inappropriateness of RNAlater to preserve *C. elegans* for RNA extraction, *Caenorhabditis elegans*, RNA extraction, RNAlater, Storage conditions, Proteinase K

## Abstract

*Caenorhabditis elegans* is a well-established laboratory animal model and has been widely used in biological research. However, it is still a challenge to obtain a good amount of quality RNA from a limited number of *C. elegans* for gene expression studies. To address this issue, the present study has compared different conditions to preserve *C. elegans* for RNA extraction after the failure of an initial effort to use RNAlater-preserved worms for RNA extraction. The effects of different concentrations of proteinase K, different worm life stages, and different worm numbers on RNA extraction were also investigated. The best results were achieved under the following conditions: 1) adult worms that were either freshly prepared or quickly frozen in liquid nitrogen followed by storage at −80 °C; 2) disruption of *C. elegans* with proteinase K (1 mg/mL) in a lysis buffer (65 °C for 10 min) prior to extraction with Trizol agent. This method can provide a stable, rapid, and effective means to extract RNA from *C. elegans* with variable worm numbers from 20 to 200.

• RNAlater was inappropriate for preserving *C. elegans* for effective RNA extraction.
• Proteinase K was verified for lysing a limited number of *C. elegans* for RNA extraction.

## Graphical abstract

**Figure:**
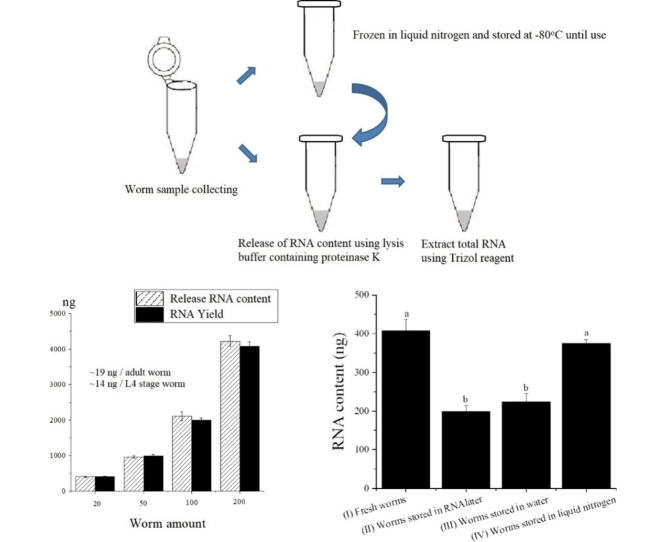

**Specification Table**

**Table:** 

Subject Area:	Agricultural and Biological Sciences
More specific subject area:	RNA extraction
Method name:	Inappropriateness of RNAlater to preserve *C. elegans* for RNA extraction
Name and reference of original method:	Ly K, Reid SJ, Snell RG. Rapid RNA analysis of individual *Caenorhabditis elegans*. MethodsX. 2015 Jan;2:59-63.
Resource availability:	NA

## Method details

*Caenorhabditis elegans*, belonging taxonomically to the pseudocoelomates of Protostomia, has a simple body structure consisting of only about 1000 cells, and has approximately 20,000 protein-coding genes [1]. However, the tissues of adult *C. elegans* possess typical cell types of all metazoans such as muscle, nerve, intestine, and skin [2]. The nematode is transparent and hermaphroditic and has a small size, short generation time, and clear genetic background. Such features are apparently advantageous for biological studies of anatomy, development, fertilization, and genetics, including mechanistic studies particularly. In the past, *C. elegans* has been used extensively as a laboratory animal model for the research in neuroscience, reproduction toxicity, aging [3,4], and interactions between microbes and hosts [5].

Transcription analysis can reveal the level of gene expression and lead to a better understanding of the physiological status of cells and/or animals. Efficient extraction of RNA to obtain a good amount and high quality of RNA remains critical in the success of transcription analysis. The traditional methods involve the use of phenol/chloroform or Trizol extraction [6]. When the samples cannot be processed for RNA extraction immediately, they are quickly frozen in liquid nitrogen and stored at −80 °C for future use. This common practice was later improved by using RNAlater that can permeate the tissue to stabilize and protect cellular RNA *in situ*, and allow the samples to be stored for a long period of time without jeopardizing the quality and quantity of RNA content [7]. Since the use of RNAlater allows large numbers of samples to be easily processed, it becomes widely used nowadays to preserve various types of cells and tissues for effective RNA extraction. *C. elegans* has resilient cuticle that needs to be ruptured prior to RNA extraction [8]. Using phenol/chloroform method to extract RNA from the nematode normally requires grinding of frozen worm samples to fine powders in the presence of liquid nitrogen before extraction [9], while freeze-thaw treatment in liquid nitrogen followed by dozens of minutes to lyse the worms at 4 °C with continuous stirring is often conducted before Trizol is used for RNA extraction [10].The resilient cuticle of fresh worms can also be ruptured by bead-beating, but the bead-beating method requires a relatively large number of worms [11]. Recently, Ly et al. reported a rapid and simple method to extract RNA from a single worm by using proteinase K [12]. However, we were unable to consistently reproduce the results with RNAlater treated *C. elegans* using the reported method (data not shown). We also increased the number of RNAlater-preserved *C. elegans* and had no success in consistently obtaining a sufficient amount of quality RNA. This has led us to investigate if the use of RNAlater is appropriate to preserve *C. elegans* for extraction of RNA from the nematode, and subsequently to develop a rapid, effective, and stable method for extracting quality RNA from a limited number of *C. elegans* (from 20 to 200 worms). The results are reported herein.

## Methods and material

*C. elegans* was cultured under standard laboratory condition [13]. Wild-type Bristol strain N2 was used and propagated at 20 °C on NGM (nematode growth medium) plates seeded with *Escherichia coli* OP50. The *C. elegans* was obtained from the *Caenorhabditis* Genetics Center (CGC), University of Minnesota, USA.

### Release of RNA content from fresh *C. elegans* using lysis buffer containing proteinase K

Adult worms collected from a NGM plate using RNase-free water and washed three times to remove bacteria were used in this experiment. To examine the effect of different proteinase K concentrations on the lysis of *C. elegans*, worms were firstly collected into a 15 mL centrifuge tube using RNAse free water, then vortex and centrifuged at 300 rpm to remove the supernatant, repeated it three times to wash the worms. 20 washed worms were transferred in 5 μL RNase-free water to a new 200 μL RNase-free Eppendorf tube, mixed with 20 μL of basic lysis buffer containing 1 or 2 mg/mL of proteinase K (Qiagen, Cat.#19133), and then incubated at 65 °C for 2, 5, or 10 min followed by 85 °C for 1 min. The basic lysis buffer was consisted of 0.5% Triton (v/v), 0.5% Tween-20 (v/v), 0.25 mM EDTA, 2.5 mM Tris-HCl buffer (pH8.0), and 8% RNAsecure (v/v; Thermo Fisher Scientific, Cat.#AM7006). Qubit RNA HS Assay kit (Thermo Fisher Scientific, Cat.#Q32852) and Qubit® 2.0 Fluorometer (Invitrogen) were used to determine the level of RNA content in the worm lysates according to instructions from the manufacturer. Proteinase K at 1 mg/mL and incubation at 65 °C for 10 min were shown to be the best to fully lyse the worms. Therefore, these optimized conditions were subsequently used in the following experiments unless indicated otherwise.

### Release of RNA content from *C. elegans* preserved under different conditions

Twenty adult worms (in 20 μL of RNAse-free water in a 2 mL Eppendof tube) stored under different conditions were used to determine total RNA content in the lysate of *C. elegans*. The storage conditions include: (I) fresh worms (no storage); (II) stored in RNAlater (Ambion, Cat.#AM7021) at 4 °C for 24 h followed by storage at −80 °C until use; (III) stored in RNase-free water without RNAlater at 4 °C for 24 h followed by storage at −80 °C until use; (IV) frozen in liquid nitrogen and then stored at −80 °C until use. The methods to lyse worms and to determine the level of RNA content in worm lysates were the same as described above.

### Extraction of RNA from *C. elegans* at different growth stages and from scaled up experiments

Twenty fresh adult worms or L4-stage worms were used to investigate the influence of growth stage over the yield of total RNA extract. After the lysis of worms to release RNA content using the optimized conditions described above, total RNA was extracted using Trizol™ Reagent according to instructions from the manufacturer (Invitrogen, Cat.# 15596026). The yield of total RNA was determined by Nano-drop 1000 Spectrophotometer (Thermo Scientific).

Scaled up experiments with different number of fresh worms were carried out to examine the efficiency of RNA extraction. Adult worms from 20 to 200 were lysed in the lysis buffer containing 1 mg/mL proteinase K based on the ratio of 1 μL lysis buffer per worm. After worm lysis using the optimized conditions, both the level of RNA content in the lysates of *C. elegans* and the yield of total RNA extracted by Trizol method were determined using the Qubit kit and fluorometer and Nano-drop 1000 Spectrophotometer, respectively.

### cDNA synthesis and quantitative PCR assays

Total RNA extracted from adult worms was treated with TURBO DNA-free™ Kit (Ambion, Cat.#AM1907) to remove DNA. One microgram of treated total RNA was used as the template for the synthesis of cDNA in a 20 μL reaction system using qScriptTM cDNA SuperMix (Quantabio, Cat.#95048-100) according to manufacturer’s protocol. One microliter cDNA template was used for quantitative PCR assays. The PCR assays were performed using a AB 7500 Real Time PCR System (Applied Biosystems Inc., Foster City, CA) with iTaq™ Universal SYBR Green Supermix (Bio-Rad, Cat.#172-5122) under the conditions as follows: 40 cycles of denaturation at 95 °C for 30 s, annealing at 56 °C for 1 min, extension at 72 °C for 30 s. The DNA sequences of PCR primers are listed in Table 1.

**Table 1:** Primer of QPCR assay.

Primer	Amplicon (bp)	Sequence (5'–3')	Source or Reference
*snb-1*F	128	CCGGATAAGACCATCTTGACG	[18]
*snb-1*R		GACGACTTCATCAACCTGAGC	
GAPDH-1F	159	ACTCGACCCACGGTCAATTC	This study
GAPDH-1R		ACTCGACAACGAAATCGGCT	
RPL-4F	183	TTGCCCGTATTCCACGTGTT	This study
RPL-4R		GGATTCCGGAGGCAGCAATA	
*daf-16*F	181	TCGTCTCGTGTTTCTCCAGC	[19]
*daf-16*R		TAATCGGCTTCGACTCCTGC	

### Statistical analysis

Results were expressed as mean ± S.D. Statistical significance of the data was performed by using one-way analysis of variance (ANOVA) in Statistical Product and Service Solutions (SPSS, Windows version 11.5), and a value of *P* ≤ 0.05 was accepted to be statistically significant.

## Results and discussion

### RNA extraction after worm lysis with proteinase K

**Fig. 1:**
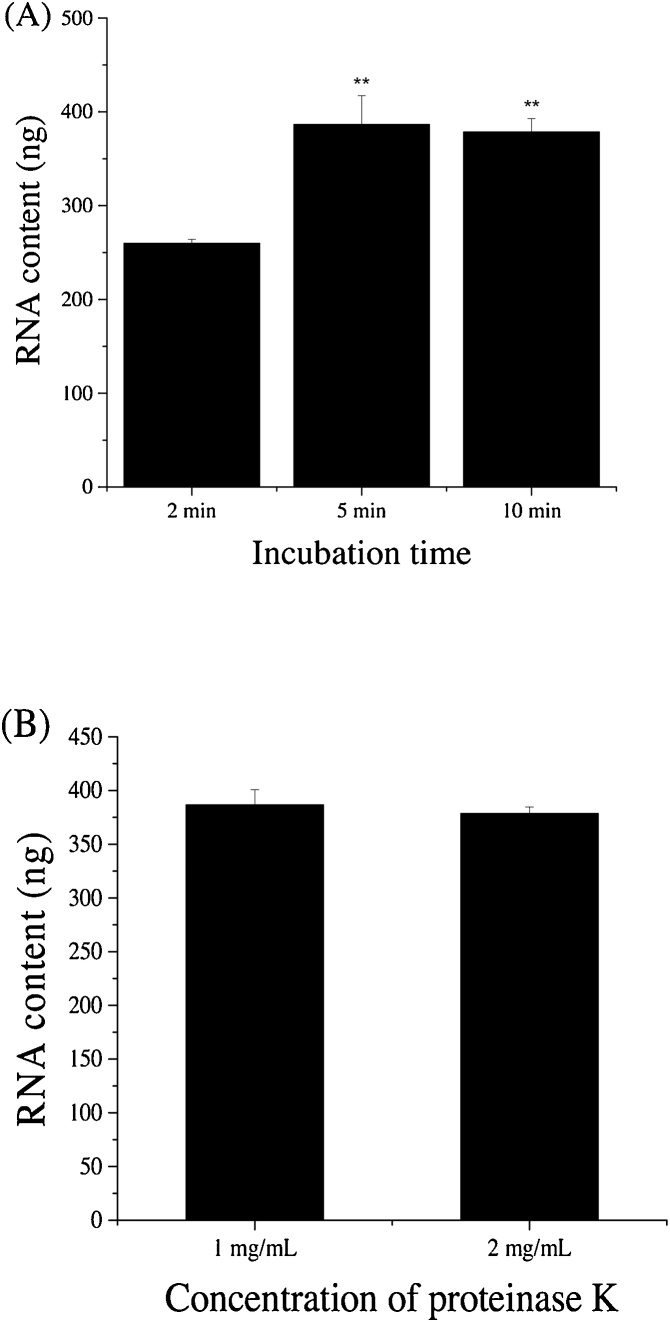
Effect of proteinase K treatments on RNA content release. (A) Effect of incubation time. (B) Effect of proteinase K concentration. Results are presented as mean ± S.D. (n = 3). **Significant difference (*P* ≤ 0.01) in the comparisons between 5-min or 10-min treatment group and 2-min group.

Freshly prepared *C. elegans* samples were firstly used to test the efficiency of proteinase K in the lysis of worms and release of RNA content. Proteinase K could effectively disintegrate *C. elegans* to release RNA for extraction in within 10 min with 20 adult worms. However, when worms were not subjected to proteinase K treatment, no worm lysis and release of RNA content were observed. When the worms were incubated in the lysis buffer containing 1 mg/mL proteinase K, 5 or 10 min incubation appeared to be sufficient to release the total RNA content (Fig. 1A). The level of released RNA content showed no significant difference between 5 and 10 min incubation (*P* > 0.05), but much higher than 2 min incubation (*P* ≤ 0.05). However, 10 min incubation generated more consistent results in RNA extraction than 5 min. When the worms were treated with proteinase K at 1 mg/mL and 2 mg/mL, respectively, no dramatic difference in worm lysis (*P* > 0.05) was observed (Fig. 1B). These data suggest that incubation of worms using basic lysis buffer containing 1 mg/mL proteinase K at 65 °C for 10 min is effective to release RNA content prior RNA extraction.

### Effects of worm storage conditions on preserving RNA

**Fig. 2:**
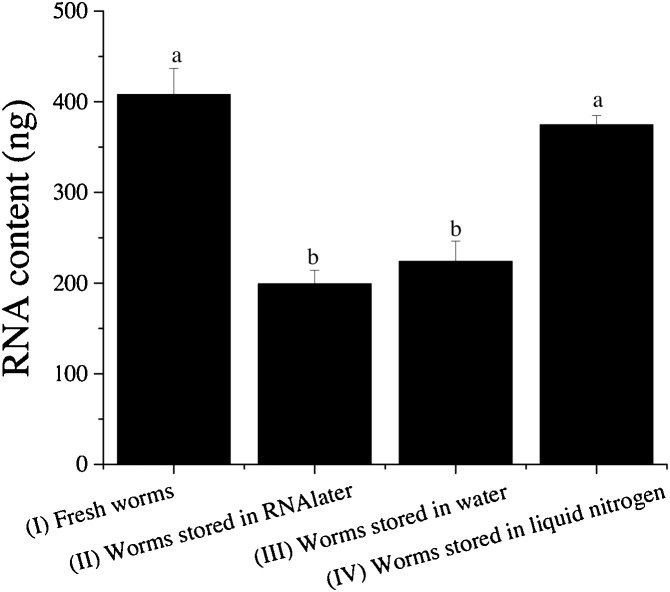
Effect of worm storage conditions on RNA yield. Four store conditions:(I) fresh worms (no storage); (II) stored in RNAlater (Ambion, Cat.#AM7021) at 4 °C for 24 h followed by storage at −80 °C until test; (III) stored in RNase-free water without RNAlater at 4 °C for 24 h followed by storage at −80 °C until test; (IV) frozen in liquid nitrogen and then stored at −80 °C until test. Results are presented as mean ± S.D. (n = 3). Different letters represent a significant difference (*P* ≤ 0.05).

As shown in Fig. 2, fresh worms without being subjected to any storage (Treatment I) appear to be the best source to obtain *C. elegans* RNA content. There was 400 ng RNA content released from 20 fresh worms. However, only a significantly reduced quantity of RNA content (∼200 ng, *P* ≤ 0.05) was obtained from the worms preserved in RNAlater at 4 °C for 24h (Treatment II), which was similar to that of worms stored in RNase-free water without RNAlater at 4 °C for 24 h (Treatment III). The worms frozen by liquid nitrogen and transferred to −80 °C immediately for storage (Treatment IV) showed no significant difference in the level of released RNA content compared to the fresh worms (*P* > 0.05).

All the results were reproducible and the RNAlater solution used for the experiment was proved functional properly when applied for extracting RNA from bacterial or Caco-2 cells. These data suggest that the storage of *C. elegans* in RNAlater cannot effectively protect *C. elegans* RNA from degradation, even though the method has widely been used to preserve various types of cells and tissues for RNA extraction [[14], [15], [16]]. To the best of our knowledge, this observation is the first report on the inappropriateness of RNAlater in preserving RNA in *C. elegans*. The low efficiency of RNAlater in preserving *C. elegans* RNA could be attributed to the possibility that RNAlater cannot effectively penetrate the tough cuticle of the nematode to inhibit RNase in the body. Further studies are required to make a conclusion.

### Yield of RNA extraction from *C. elegans* at different life stages and from the scale up experiments

**Fig. 3:**
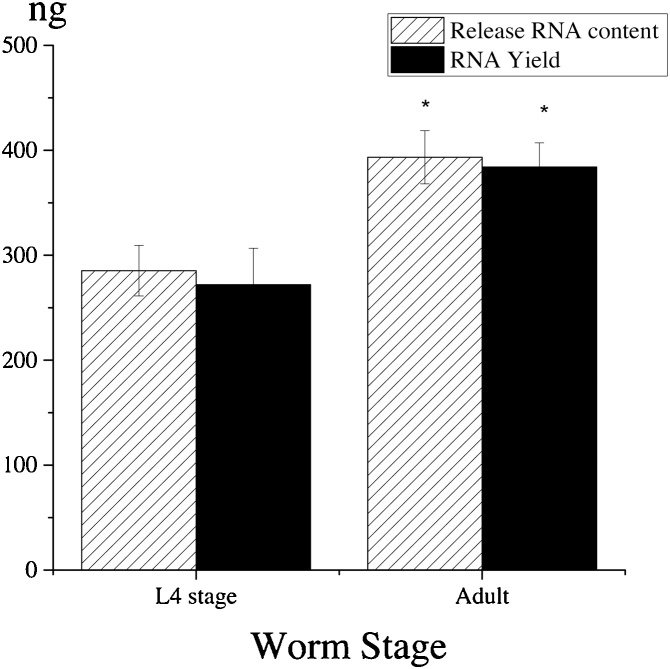
RNA extract from worms at different life stage. Results are presented as mean ± S.D. (n = 3). *Significant difference (*P* ≤ 0.05) in the comparisons between L4-stage and adult worms within each treatment.

As shown in Fig. 3, 393.33 ± 23.32 ng RNA content was obtained from the lysate of 20 fresh adult worms, while the lysate of 20 fresh L4-stage worms contained 285.33 ± 24.11 ng RNA content. The average RNA content from each worm is therefore approximately 19.65 ng for adult worms and 14.25 ng for L4-stage worms. After extraction of the lysates by Trizol method, the RNA yield from the adult worms was 384.00 ± 23.07 ng, while the yield from the L4-stage worms by the same method was 272.00 ± 34.70 ng, which was equal to approximately 19.2 ng per adult worm and 13.6 ng per L4-stage worm. These data indicate that the total RNA in the lysates generated by proteinase K treatment can be effectively extracted using Trizol method. Besides, a significantly higher RNA yield can be obtained from adult worms than from L4-stage worms.

**Fig. 4:**
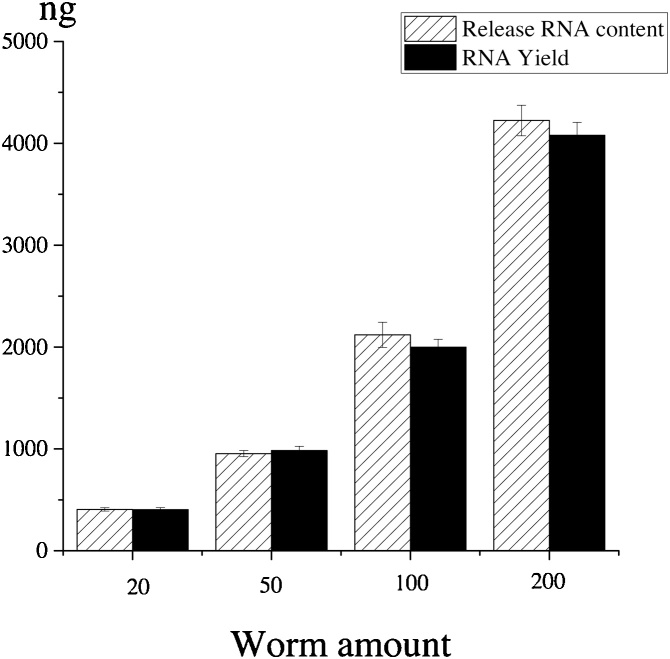
RNA extract from different numbers of worms. Results are presented as mean ± S.D. (n = 3). There is a linear relationship (y = 20.48x − 27.88, R^2^ = 0.9976) between worm numbers and the yield of RNA extract. There is a correlation between the released RNA content after worm lysis measured by Qubit kit/fluorometer and RNA yield determined by Nano-drop 1000 Spectrophotometer after RNA extraction (Pearson correlation test: r = 0.9989, P < 0.0001).

The results of the scale-up experiments are shown in Fig. 4. From 20 adult worms, 404.00 ± 20.30 ng of total RNA was obtained (approximately 20.2 ng per adult). When the worm number reached 200, the total RNA extract was increased to 4079.67 ± 126.43 ng (approximately 20.39 ng per worm). There appeared to be a linear relationship between worm numbers and the yield of RNA extract. In addition, the total RNA amount obtained by the Trizol extraction from each sample was similar to that determined by the Qubit kit/fluorometer method. Thus, the method developed in the present study can be effectively used to extract RNA from not only a limited, but also a larger number of worms. Ly et al. reported recently that a single fresh adult worm contained about 35 ng of total RNA [12]. In the present study, the average of total RNA extract per frozen adult worm was approximately 20 ng. It is unclear at present if the difference was caused by using fresh *versus* frozen worm samples.

### RNA quality

The total RNA extract after removal of DNA was applied to real-time PCR assays to determine the quality of the RNA extract. The C_t_ values of three housekeeping genes from the 20-worm group, including *snb-1*, GAPDH, and RPL-4, and gene *daf-16* that is a member in the insulin/insulin-like growth factor pathway of the nematode [17], were 22.56 ± 0.13, 23.77 ± 0.26, 20.65 ± 0.07,and 24.24 ± 0.08, respectively. Additionally, the results of quantitative PCR assays from the 50-worm and 100-worm group were similar to those of the 20-worm group. Thus, the quality of total RNA extract from both the small and large number of worms appears to be good and can warrant PCR assays.

## Conclusion

The commonly used method with RNAlater to preserve various types of cells and tissues for RNA extraction doesn’t appear to be appropriate for preserving *C. elegans* for effective RNA extraction. The method described in the present study that uses proteinase K to lyse the nematode offers an additional choice for rapid, simple, and consistent RNA extraction from a limited number of *C. elegans*.

## Declaration of Competing Interest

None of the authors of this study has any financial interest or conflict with industries.

## References

[1] Stein L.D., Bao Z., Blasiar D., Blumenthal T., Brent M.R., Chen N., Chinwalla A., Clarke L., Clee C., Coghlan A., Coulson A., D’Eustachio P., Fitch D.H.A., Fulton L.A., Fulton R.E., Griffiths-Jones S., Harris T.W., Hillier L.W., Kamath R., Kuwabara P.E., Mardis E.R., Marra M.A., Miner T.L., Minx P., Mullikin J.C., Plumb R.W., Rogers J., Schein J.E., Sohrmann M., Spieth J., Stajich J.E., Wei C., Willey D., Wilson R.K., Durbin R., Waterston R.H. (2003). The genome sequence of Caenorhabditis briggsae: a platform for comparative genomics. PLoS Biol..

[2] Spencer W.C., Zeller G., Watson J.D., Henz S.R., Watkins K.L., McWhirter R.D., Petersen S., Sreedharan V.T., Widmer C., Jo J., Reinke V., Petrella L., Strome S., Von Stetina S.E., Katz M., Shaham S., Ratsch G., Miller D.M. (2011). A spatial and temporal map of C. elegans gene expression. Genome Res..

[3] Li Y., Gao S., Jing H., Qi L., Ning J., Tan Z., Yang K., Zhao C., Ma L., Li G. (2013). Correlation of chemical acute toxicity between the nematode and the rodent. Toxicol. Res..

[4] Asthana J., Yadav A.K., Pant A., Pandey S., Gupta M.M., Pandey R. (2015). Specioside ameliorates oxidative stress and promotes longevity in Caenorhabditis elegans. Comp. Biochem. Physiol. Part C: Toxicol. Pharm..

[5] Ewbank J. (2003). The nematode Caenorhabditis elegans as a model for the study of host-pathogen interactions. Journal De La Société De Biologie.

[6] Burdine R., Stern M. (1996). Easy RNA Isolation from C. elegans: A TRIZOL Based Method.

[7] Mutter G.L., Zahrieh D., Liu C., Neuberg D., Finkelstein D., Baker H.E., Warrington J.A. (2004). Comparison of frozen and RNALater solid tissue storage methods for use in RNA expression microarrays. BMC Genomics.

[8] Bhaskaran S., Butler J.A., Becerra S., Fassio V., Girotti M., Rea S.L. (2011). Breaking Caenorhabditis elegans the easy way using the Balch homogenizer: an old tool for a new application. Anal. Biochem..

[9] Ketting R.F., Tijsterman M., Plasterk R.H.A. (2006). Isolation of RNA from C. elegans. Cold Spring Harb. Protoc..

[10] Greer E.L., Dowlatshahi D., Banko M.R., Villen J., Hoang K., Blanchard D., Gygi S.P., Brunet A. (2007). An AMPK-FOXO pathway mediates longevity induced by a novel method of dietary restriction in C. elegans. Curr. Biol..

[11] Zhou M., Yu H., Yin X., Sabour P.M., Chen W., Gong J. (2014). Lactobacillus zeae protects Caenorhabditis elegans from enterotoxigenic Escherichia coli-caused death by inhibiting enterotoxin gene expression of the pathogen. PLoS One.

[12] Ly K., Reid S.J., Snell R.G. (2015). Rapid RNA analysis of individual Caenorhabditis elegans. MethodsX.

[13] T. Stiernagle, WormBook, 11 February 2006.

[14] Grotzer Michael A., Patti R., Geoerger B., Eggert A., Chou Thomas T., Phillips Peter C. (2000). Biological stability of RNA isolated from RNAlater-treated brain tumor and neuroblastoma xenografts. Med. Pediatr. Oncol..

[15] Gorokhova E. (2005). Effects of preservation and storage of microcrustaceans in RNAlater on RNA and DNA degradation. Limnol. Oceanogr. Methods.

[16] Medeiros M., Sharma V.K., Ding R., Yamaji K., Li B., Muthukumar T., Valderde-Rosas S., Hernandez A.M., Muñoz R., Suthanthiran M. (2003). Optimization of RNA yield, purity and mRNA copy number by treatment of urine cell pellets with RNAlater. J. Immunol. Methods.

[17] Murphy C.T., McCarroll S.A., Bargmann C.I., Fraser A., Kamath R.S., Ahringer J., Li H., Kenyon C. (2003). Genes that act downstream of DAF-16 to influence the lifespan of Caenorhabditis elegans. Nature.

[18] Irazoqui J.E., Urbach J.M., Ausubel F.M. (2010). Evolution of host innate defence: insights from Caenorhabditis elegans and primitive invertebrates. Nat. Rev. Immunol..

[19] Zhou M., Liu X., Yu H., Yin X., Nie S.P., Xie M.Y., Chen W., Gong J. (2018). Cell signaling of Caenorhabditis elegans in response to enterotoxigenic Escherichia coli infection and Lactobacillus zeae protection. Front. Immunol..

